# Development, validation, and results of a survey to measure understanding of cardiopulmonary resuscitation choices among ICU patients and their surrogate decision makers

**DOI:** 10.1186/1471-2253-14-15

**Published:** 2014-03-08

**Authors:** Michael E Wilson, Abbasali Akhoundi, Artur K Krupa, Richard F Hinds, John M Litell, Ognjen Gajic, Kianoush Kashani

**Affiliations:** 1 Department of Medicine, Division of Pulmonary and Critical Care Medicine, Mayo Clinic, 200 First Street SW, Rochester, MN 55905, USA; 2 Department of Medicine, Divisions of Nephrology and Critical Care Medicine, Mayo Clinic, Rochester, MN 55905, USA; 3 Department of Critical Care Medicine, University of Hawaii, John A. Burns School of Medicine, Honolulu, Hawaii, USA; 4 Anesthesia Critical Care Research Unit, Mayo Clinic, Rochester, MN 55905, USA; 5 Divisions of Emergency and Critical Care Medicine, Beth Israel Deaconess Medical Center, Boston, Massachusetts, USA

**Keywords:** Cardiopulmonary resuscitation, Questionnaires, Health knowledge, Intensive care unit

## Abstract

**Background:**

Shared-decision-making about resuscitation goals of care for intensive care unit (ICU) patients depends on a basic understanding of cardiopulmonary resuscitation (CPR). Our objective was to develop and validate a survey to assess comprehension of CPR among ICU patients and surrogate decision-makers.

**Methods:**

We developed a 12-item verbally-administered survey incorporating input from patients, clinicians, and expert focus groups.

**Results:**

We administered the survey to 32 ICU patients and 37 surrogates, as well as to 20 resident physicians to test discriminative validity. Median (interquartile range) total knowledge scores were 7 (5-10) for patients, 9 (7-12) for surrogates, and 14.5 (14-15) for physicians (p <.001). Forty-four percent of patients and 24% of surrogates could not explain the purpose of CPR. Eighty-eight percent of patients and 73% of surrogates could not name chest compressions and breathing assistance as two components of CPR in the hospital. Forty-one percent of patients and 24% of surrogates could not name a single possible complication of CPR. Forty-three percent of participants could not specify that CPR would be performed with a full code order and 25% of participants could not specify that CPR would not be performed with a do-not-resuscitate order. Internal consistency (Cronbach’s alpha = 0.97) and test-retest reliability (Pearson correlation = 0.96, p < .001) were high.

**Conclusions:**

This easily administered survey, developed to measure knowledge of CPR and resuscitation preference options among ICU patients and surrogates, showed strong face validity, content validity, internal consistency, test-retest reliability, and discriminative validity. A substantial proportion of ICU patients and surrogates decision-makers have poor knowledge of CPR and basic resuscitation options.

## Background

Health care providers should discuss cardiopulmonary resuscitation (CPR) preferences with patients who are at risk of requiring CPR, in order to ensure that this intervention is in accordance with the patient’s goals of care [1,2]. Nevertheless, such code status discussions occur with varying frequency, even for hospitalized and critically ill patients [3-6]. Discussions regarding resuscitation preferences can be difficult and confusing for patients, surrogates, and providers [7]. While conversations about resuscitation preferences should optimally occur prior to the development of critical illness, this is often not the case and discussions occur in the context of acute critical illness and emotional distress. In addition, discussions about CPR are often unnecessarily obscured by medical jargon and do not contain the elements suggested by professional societies and bioethicists [8].

Knowledge of CPR and resuscitation choices is one key component of shared medical decision making [9]. While previous survey instruments have been utilized to measure knowledge of CPR components and success rates [3,4,10-16], their measurement of resuscitation choices and other CPR-related terminology, as well as validation and testing in intensive care unit (ICU) populations, is limited. For this study our objectives were to: 1) Develop and validate a survey to measure patient and surrogate decision-maker understanding of resuscitation terminology and resuscitation options, and 2) Use the validated survey to measure the understanding of resuscitation terminology and resuscitation options in a cohort of ICU patients and surrogates. We hypothesized that patients and surrogates would have limited knowledge of CPR and CPR choices in the hospital, and that surrogates’ understanding would be comparatively better.

## Methods

### Survey development

**Figure 1:**
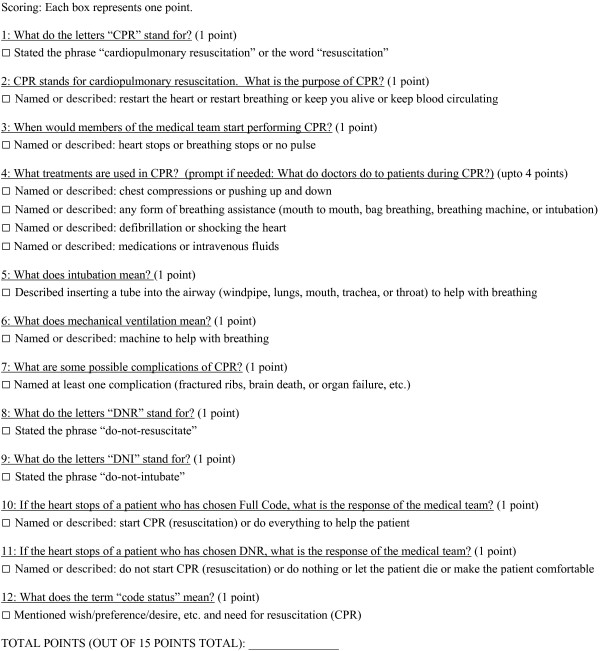
CPR survey instrument (with criteria for receiving credit for a given response).

A list of possible survey items regarding CPR and resuscitation preferences was generated from interviews with patients, surrogates, internal medicine resident physicians, ICU nurses, ICU attending physicians, palliative care physicians, patient education specialists, as well as a literature review of existing resuscitation surveys [3,4,10-16]. This comprehensive list was then refined based on item content and usability by expert consensus of a group of five ICU physicians, five ICU nurses, and three patient education specialists. This group determined that, due to critical illness, ICU patients and their surrogates would best be served by a verbally administered questionnaire. The survey instrument assessed knowledge of the possible components of CPR in the hospital, the definition of CPR related terms and acronyms, as well as the various resuscitation preference options for hospitalized patients (see Figure 1). The survey assessed patients’ core level of knowledge of CPR (such as “What is the purpose of CPR” and “What treatments are used in CPR?”) as well as the meaning of commonly used medical terms (such as “What does intubation mean?”). The survey consisted of 12 questions with one point being awarded for each correct response. Question four had a total of four possible correct answers. Thus the score survey score ranged from 0–15 points, with higher scores representing increased knowledge.

### Face and content validity

Face validity (the extent to which the survey appeared to measure its intended domain) and content validity (the extent to which the survey measured all aspects of its intended domain) were assessed via two thirty minute focus group sessions with ICU physician and nurse participants. The focus groups assessed the items for accuracy, clarity, relevance, completeness, breadth, and usability in an ICU population. We then pilot tested this survey in a small group of ten ICU patients and ten surrogates, recording their answers and any misunderstandings about the questions. We then modified the survey questions based on this feedback.

### Discriminative validity

Discriminative validity (the extent to which the survey distinguished between two groups of subjects who were expected to perform differently) was measured by comparing the survey results of the patients and surrogate cohorts to a cohort of internal medicine resident physicians, who were expected to demonstrate an increased knowledge.

### Test-retest reliability

Test-retest reliability (the extent to which survey results were similar under different conditions or periods of time) was measured by repeating the survey in patients and surrogates 24 hours after the initial survey. Physician surveys were repeated two weeks apart.

### Study participants

The revised survey (see Figure 1) was then verbally administered verbatim by a single survey administrator to patients and surrogates in one surgical ICU and one mixed medical/surgical ICU in a single medical center. The survey administrator was trained in survey administration and result interpretation. The survey administrator approached the medical team (physicians and nurses) of consecutive patients admitted to the ICUs in the previous 48 hours. If the medical team determined that patients were making their own medical decisions and were not delirious, then patients were approached to enroll in the study. Patient orientation to month, year, and place was then objectively verified and informed consent was obtained. If patients with decision making capacity were unavailable to participate (such as away at testing) on two occasions, then they were excluded from the study. If patients were disoriented or were assessed not to be making their own medical decisions, then the patients’ surrogate decision makers (as named by the medical team) were approached to enroll in the study. Excluded groups included minors, prisoners, pregnant women, and non-English speakers. For the purpose of testing discriminative validity, the survey was also administered to 20 internal medicine resident physicians after informed consent was obtained. The Mayo Clinic Institutional Board of Review approved the study protocol.

### Statistical analysis

Paired Student’s t-tests, the Wilcoxon’s rank-sum test, and the chi-square test were used as appropriate for univariate comparisons. Test-retest reliability was performed using a Pearson correlation for total knowledge scores. Internal consistency was measured using Cronbach’s alpha. P values < 0.05 were considered statistically significant. Statistical analysis was performed with JMP (JMP, Version 9, SAS Institute Inc.).

## Results

**Figure 2:**
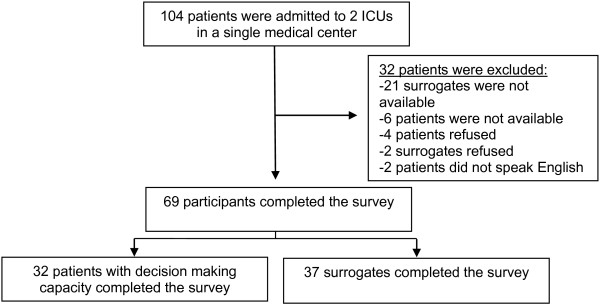
Study enrollment.

One hundred four ICU patients were approached for participation in the study, of which thirty two met exclusion criteria (Figure 2). In total, the survey was verbally administered to 32 patients and 37 surrogate decision members, as well as to 20 internal medicine residents test discriminative validity (Table 1).

**Table 1:** Characteristics of participants

**Characteristic**	**Patients (n = 32)**	**Surrogates (n = 37)**	**Physicians (n = 20)**
Age, years, median (IQR)	62(50–71)	49(41–63)	29(27–32)
Female gender, n (%)	8(25)	23(62)	7(35)
Non-Caucasian race, n (%)	0(0)	4(11)	9(45)
Marital status, n (%)			
Single	2(6)	7(20)	11(55)
Married	23(72)	25(71)	9(45)
Widowed	4(13)	0(0)	0(0)
Divorced	3(9)	3(9)	0(0)
Education, n (%)			
Eighth grade or less	0(0)	1(3)	0(0)
High school	7(22)	9(24)	0(0)
Some college	10(31)	12(32)	0(0)
College graduate	5(16)	12(32)	0(0)
Postgraduate	6(19)	3(8)	20(100)
Unknown	4(12)	0(0)	0(0)
Religious preference, n (%)			
Christian, non-Catholic	22(69)	16(44)	10(50)
Catholic	10(31)	11(31)	4(20)
Jewish	0(0)	1(3)	0(0)
Muslim	0(0)	0(0)	1(5)
Other	0(0)	3(8)	2(10)
None	0(0)	5(14)	3(15)
Self-reported health status, n (%)			
Excellent	2(6)	7(19)	10(50)
Very good	3(9)	17(46)	9(45)
Good	4(13)	11(30)	1(5)
Fair	11(34)	2(5)	0(0)
Poor	12(38)	0(0)	0(0)
Health literacy, n (%)			
Extreme	13(41)	17(46)	14(70)
Quite a bit	5(16)	11(30)	5(25)
Somewhat	6(19)	4(11)	1(5)
A little bit	3(9)	1(3)	0(0)
Not at all	5(16)	4(11)	0(0)
Prior health care experience, n (%)	6(19)	14(38)	20(100)
Has previously performed CPR on somebody else, n (%)	2(6)	4(11)	19(95)
Has had CPR previously performed on themselves, n (%)	2(6)	2(5)	0(0)
Medical patient, n (%)	18(56)	14(44)	NA
Surgical patient, n (%)	27(73)	10(27)	NA
ICU admission APACHE III score, median (IQR)	53(44–71)	53(49–72)	NA
Code status on ICU admission, n (%)			NA
Full code	29(91)	37(100)	NA
DNR	3(9)	0(0)	NA
ICU length of stay, median (IQR)	2(1–3)	3(2–9)	NA
Hospital length of stay, median (IQR)	6(3–14)	14(6–21)	NA
Surrogate relationship to patient, n (%)	NA		NA
Spouse or partner	NA	15(41)	NA
Parent	NA	3(8)	NA
Child	NA	14(38)	NA
Sibling	NA	2(5)	NA
Other	NA	3(8)	NA

IQR, interquartile range; CPR, cardiopulmonary resuscitation; ICU, intensive care unit; DNR, do not resuscitate.

### Survey results and discriminative validity

Median (interquartile range) total knowledge scores were 7 (5-10) out of 15 for patients, 9 (7-12) for surrogates, and 14.5 (14-15) for physicians with a p value of <0.001, representing excellent discriminative validity (Table 2).

**Table 2:** CPR knowledge scores and discriminative validity

**Question (%)**	**Patients (n = 32)**	**Surrogates (n = 37)**	**Physicians (n = 20)**	**P value**
What do the letters CPR stand for?	41%	57%	90%	.002
What is the purpose of CPR?	56	76	100	.002
When would members of the medical team start performing CPR?	75	84	95	.17
What treatments are used in CPR?				
Chest compressions	81	92	90	.38
Breathing assistance	56	76	95	.008
Defibrillation	23	35	75	<.001
Medications or fluids	9	24	90	<.001
Could correctly identify chest compressions and breathing assistance as components of CPR	12	27	85	<.001
What does intubation mean?	37	57	100	<.001
What does mechanical ventilation mean?	34	68	100	<.001
What are some possible complications of CPR?	59	76	100	.004
What do the letters “DNR” stand for?	41	59	100	<.001
What do the letters “DNI” stand for?	34	49	100	<.001
Response to full code	63	51	100	<.001
Response to DNR	69	81	100	.02
What does code status mean?	22	8	95	<.001
Total knowledge score, 0–15 points (IQR)	7(5–10)	9(7–12)	14.5(14–15)	<.001

CPR, cardiopulmonary resuscitation; DNR, do not resuscitate; DNI, do not intubate; IQR interquartile range.

Fifty six percent of patients and 76% of surrogates could explain the purpose of CPR. Only 12% percent of patients and 27% of surrogates could name chest compressions and breathing assistance as components of CPR. Forty one percent of patients and 24% of surrogates could not name a single possible complication of CPR. Similarly, 37% of patients and 49% of surrogates were unable to conclude that CPR would be performed if the patient chose to have a Full Code status and 31% of patients and 19% of surrogates were unable to conclude that CPR would not be performed if the patient chose to have a Do Not Resuscitate code status. Two out of 20 internal medicine residents did not answer correctly the question “What do the letters CPR stand for?” and one out of 20 residents who had just started his/her training had not previously performed CPR.

Factors associated with lower knowledge scores for patients and surrogates include: advanced age, male sex, widowed or divorced marital status, and lower education level. Patients with lower self-perceived health literacy and lack of prior health care experience also had lower total knowledge scores. There was no observed association between total knowledge scores and race, prior experience performing CPR, primary ICU service, Acute physiology and chronic health evaluation III (APACHE III) score, or admission code status order (Table 3).

**Table 3:** Predictors of higher knowledge scores in patients and surrogates

**Characteristic**	**Total knowledge score, median (IQR)**	**p value**
Age, years		.03
≥ 60	7(5–9)	
< 60	9(7–12)	
Sex		.05
Female	9(7–12)	
Male	7(5–10)	
Race		.23
Non-Caucasian race	8(5–10)	
Caucasian	10(8–12)	
Marital status		.02
Single	10(8–13)	
Married	8(5–10)	
Widowed	5.5(2–6)	
Divorced	7(5–8)	
Education		.03
≥ college graduate	7(5–10)	
< college graduate	10(7–12)	
Self-reported health status		.07
Fair or poor	7(5–10)	
Good or better	9(7–12)	
How confident are you in filling out medical forms by yourself? (health literacy)		.01
Somewhat confident or less	7(3–9)	
Extremely or quite a bit confident	9(7–11)	
Prior health care experience		.002
Yes	10(7–13)	
No	7(5–10)	
Have you performed CPR on somebody else?		.17
Yes	11(7–13)	
No	8(5–10)	
Has CPR been performed on you?		.03
Yes	5(2–7)	
No	8(6–11)	
Primary ICU service		.84
Medical patient	7(5–12)	
Surgical patient	8(6–10)	
ICU admission APACHE III score		.19
≥ 70	7(4-10	
< 70	8(7–11)	
Code status on ICU admission		.27
Full code	8(6-10	
DNR	6(2–10)	

IQR, interquartile range; CPR, cardiopulmonary resuscitation; ICU, intensive care unit; DNR, do not resuscitate.

### Internal consistency and test-retest reliability

Cronbach’s alpha for the total knowledge score was 0.97, with values > 0.7 representing acceptable internal consistency. Test-retest reliability was performed on 36 study participants. The correlation between pretest and posttest total knowledge scores was high with a Pearson correlation of 0.96 with a 95% confidence interval of 0.92-0.99 (p < .001).

## Discussion

This survey was developed to measure CPR knowledge in critically ill patients and their surrogate decision makers. The survey showed strong face and content validity, as well as internal consistency, test-retest reliability, and discriminative validity. The survey was easily administered to a cohort of ICU patients and surrogates by a survey administrator who read the survey verbatim. Initial survey results showed that patients and surrogate decision makers had relatively poor knowledge of CPR terminology, components, complications, and available preference options. While our results confirm prior studies that have shown patients’ limited understanding of the definition of CPR and its components [3,4,10-16], our survey showed these results in a validated format in an ICU population and incorporated additional questions to extend the assessment of knowledge of resuscitation preferences (ie Full Code, Do Not Resuscitate, and Do Not Intubate).

In the hospital, patient instruction regarding CPR and resuscitation preference options occurs primarily during code status discussions. These circumstances of these discussions–often brief, laden with medical jargon, occurring under stressful circumstances with providers at various levels of training–may actually contribute to poor knowledge among patients and their families [8]. Furthermore, code status discussions also occur with varying frequency [3-6] and contain variable content [17]. The impact of critical illness, age, and patients’ perceived health literacy may also contributed to limited comprehension. Additionally, some patients may avoid discussing CPR with their health care providers, delaying complex decision making and potentially impairing knowledge acquisition [4]. It should be noted that code status discussions should not occur in isolation, and are part of a larger assessment of the patients’ preferences, values, and goals of medical treatment. Discussions about resuscitation preferences should ideally occur as part of advance care planning in the outpatient setting.

As we hypothesized, surrogate decision makers had somewhat higher total knowledge scores than did patients. Potential reasons to explain this observed difference include, surrogates were younger, predominantly female, had more health care experience, and had greater perceived health and self-reported health literacy. Although having a family member hospitalized in the ICU has been associated with high rates of psychologic distress and burnout among surrogate decision makers [18,19] our survey was not designed to determine if this impacted surrogates’ knowledge scores. In addition, cognitive factors such as emotional distress, pain, anxiety, and depression in ICU patients with decision making capacity may impact decision making about complex and sensitive issues such as resuscitation preferences.

Although nearly all internal medicine resident physician participants answered every survey question correctly, some physicians did not know what the letters CPR stood for, could not name all of the components of CPR in the hospital, or had not previously performed CPR on a patient. These findings reveal that some physicians in training have a degree of unfamiliarity with CPR. In a system where a majority of code status discussions occur between patients and physicians in training, physician unfamiliarity may impact patient knowledge and decision making [20]. Code status discussions should occur with clinicians who have received sufficient training and experience in resuscitation decision making.

Our study has several limitations. The survey instrument did not attempt to measure respondents’ understanding of CPR survival rates, which has been shown to be an important factor in patient and surrogate CPR decision making [6,12,13,15]. We did not control for participant recollection of occurrence or content of CPR discussions with health care providers, which may have impacted knowledge scores. We did not control for socioeconomic status. Patient race has been shown to introduce variability in ICU decision making, and our study participants were mostly Caucasian and entirely English speaking [21]. We also did not measure patient/surrogate satisfaction with the survey. The survey was tested in a single center with a limited number of participants. Additionally, it was only tested in internal medicine residents, and not in a larger population of physicians at various stages in their careers.

According to the current prevailing paradigm of patient-centered care, treatment decisions are ideally made using a shared decision-making model between patients, their surrogate decision makers, and their medical providers. Patient education regarding CPR and available CPR options is an essential step in this shared decision making process. This study confirms that patients and surrogates have a limited understanding of CPR in the hospital and highlights the need to develop interventions that can improve CPR knowledge and decision making, especially since prior interventions such as information leaflets have shown limited impact [22,23].

## Conclusions

A verbally administered survey to measure CPR knowledge among critically ill patients and their surrogate decision makers showed strong face and content validity, as well as internal consistency, test-retest reliability, and discriminative validity in an ICU population. Results from our initial survey administration showed relatively poor knowledge of CPR as well as CPR preference options among both ICU patients and their surrogates. This survey instrument can be used in intervention studies seeking to improve knowledge of CPR and CPR resuscitation choices in the ICU.

## Key messages

• ICU patients and surrogates have poor understanding of basic resuscitation choices and knowledge of CPR.

• Our validated survey can be utilized in future studies to measure to assess patient and surrogate understanding of CPR and resuscitation choices.

## Abbreviations

CPR: Cardiopulmonary resuscitation; ICU: Intensive care unit; APACHE III: Acute physiology and chronic health evaluation III; DNR: Do not resuscitate; DNI: Do not intubate.

## Competing interests

The authors declare that they have no competing interests.

## Authors’ contributions

MW participated in study design, survey distribution, statistical analysis, and drafted the manuscript. AK and RH participated in survey distribution, study coordination, and statistical analysis. JL participated in study design and manuscript preparation. OG and KK participated in study design, oversight, and manuscript review. All authors read and approved the final manuscript.

## Pre-publication history

The pre-publication history for this paper can be accessed here:

http://www.biomedcentral.com/1471-2253/14/15/prepub
